# Digitale Zukünfte der Universität: Szenarien soziotechnischen Wandels

**DOI:** 10.1007/s11614-022-00507-x

**Published:** 2022-12-07

**Authors:** Bernhard Wieser, Mia Bangerl, Kübra Karatas

**Affiliations:** 1 STS-Unit am Institute of Interactive Systems and Data Science, Technische Universität Graz, Inffeldgasse 16c, 8010 Graz, Österreich; 2 Know-Center GmbH – Research Center for Data-Driven Business & Big Data Analytics, Inffeldgasse 13, 8010 Graz, Österreich; 3 Institut für Soziologie, Karl-Franzens-Universität Graz, Universitätsstraße 15/G4, 8010 Graz, Österreich

**Keywords:** Digitalisierung, Soziotechnischer Wandel, MLP, COVID-19, Digitale Universität, Digitization, Sociotechnical change, MLP, COVID-19, Digital university

## Abstract

Für die Universität erwiesen sich digitale Technologien während der COVID-19-Pandemie als zentrales Element der Krisenbewältigung. Das gilt insbesondere für den Bereich der Lehre. Aus Sicht der „Multi-Level-Perspektive“ (Geels 2004) eröffnen die disruptiven Auswirkungen der Pandemie ein „Window of Opportunity“ für einen tiefgreifenden und dauerhaften soziotechnischen Wandel. Daran anschließend wird in diesem Artikel die Frage erörtert, wie Angehörige der Universität die zukünftige Bedeutung digitaler Technologien einschätzen. Auf Basis qualitativer, empirischer Erhebungen lassen sich fünf Szenarien herausarbeiten, die in je unterschiedlicher Weise digitale Zukünfte skizzieren. Die Analyse dieser Zukunftsszenarien fragt jedoch nicht nach deren Eintrittswahrscheinlichkeit, sondern nach ihrer Wünschbarkeit. Auf diese Weise kann gezeigt werden, mit welchen Begründungen argumentiert wird, warum die weiterführenden Digitalisierungsschritte an der Universität unternommen oder auch unterlassen werden sollten. Zum gegebenen Zeitpunkt ist es nicht einschätzbar, welche Szenarien digitaler Universitäten sich letztlich durchsetzen werden. Nicht zuletzt deshalb versteht sich dieser Artikel als Grundlage für eine breite Debatte, die es erst zu führen gilt.

## Abstract (en)

For the university, digital technologies proved to be a central element of crisis management during the COVID-19 pandemic. This is especially true for teaching. From a “multi-level perspective” (Geels 2004), the disruptive effects of the pandemic open a “window of opportunity” for profound and lasting sociotechnical change. Against this backdrop, this article discusses how members of the university assess the future significance of digital technologies. On the basis of qualitative, empirical research, five scenarios can be distinguished, each of which outlines digital futures in a different way. However, with our analysis of these future scenarios we do not scrutinise the probability of their occurrence, but their desirability. In this way, we identify justifications diploid to argue why further steps towards a digital university should be taken or why not. At this point in time, it is impossible to assess which scenarios of digital universities will ultimately prevail. Not least for this reason, this article is intended as a basis for a broad debate that is yet to be conducted.

## Einleitung

Im universitären Kontext wurde der Umgang mit COVID-19 als beträchtlicher Digitalisierungsschub erlebt. Plötzlich war der Zutritt zum Campus für Universitätsangehörige nicht mehr möglich. In dieser Situation ging es vor allem darum, den Betrieb an der Universität aufrechtzuerhalten. Zweifelsohne waren die Herausforderungen für den Bereich der Lehre besonders groß (vgl. etwa Bork-Hüffer et al. 2021; Gurukkal 2020; Horváth et al. 2021). Der Einsatz digitaler Lösungen zur Durchführung der Lehre im Distanzmodus war daher essenziell für die Bewältigung der Krise (vgl. Vargo et al. 2021, S. 19). In welcher Weise digitale Technologien langfristig in die universitäre Lehre integriert werden, ist jedoch weitgehend unklar geblieben (vgl. Zhu und Liu 2020, S. 698; siehe auch Neuwirth et al. 2020, S. 3).

Obgleich digitale Technologien als effektive Instrumente der Krisenbewältigung anerkannt wurden, sehen darin nur sehr wenige ein Modell für die Zukunft. In der aktuellen Literatur wurde auf die Unzufriedenheit vieler Universitätsangehöriger hingewiesen (vgl. Ewing 2021, S. 41). Digitale Unterrichtsformen werden mit höherem Aufwand für Lehrende (Horváth et al. 2021, S. 9) sowie unvermeidbaren Qualitätsminderungen (Gurukkal 2020, S. 93 f.) assoziiert. Allerdings wurde auch das prä-pandemische Präsenzuniversitätsmodell als unzureichend beurteilt (Eringfeld 2021, S. 147). Viele Autor*innen betrachten die Pandemie als Entwicklungschance (z. B. Arnove 2020, S. 44 f.; Neuwirth et al. 2020, S. 3) – Dumulescu et al. sprechen in diesem Zusammenhang sogar von einer „digital revolution“ (2021, S. 1). Eine klare Richtung oder Ziele der angestrebten universitären Zukunft sind bislang kaum zu erkennen. Lübcke et al. erklären den Mangel an konkreten Zukunftsmodellen damit, dass „einige Hochschulen weiterhin im Krisenbewältigungsmodus sind und noch nicht in einer Phase strategischer Weichenstellungen“ (Lübcke et al. 2022, S. 6).

Die Autor*innen dieses Textes nehmen diese Situation zum Anlass, den beobachteten Digitalisierungsschub im Kontext der COVID-19-Pandemie als soziotechnischen Wandel (vgl. Geels 2004) zu analysieren. Insbesondere fragen wir nach den Bedingungen einer dauerhaften Etablierung des durch die Pandemie induzierten Digitalisierungsschubes. Wir vertreten die These, dass aus einer Multi-Level-Perspektive (Geels 2002, 2004; Geels und Schot 2007) die Voraussetzungen für einen nachhaltigen soziotechnischen Wandel der universitären Lehre vorerst nur teilweise gegeben sind und maßgebliche Elemente bislang fehlen. Der vorliegende Artikel versucht, über die vorherrschende Auseinandersetzung mit krisengetriebenen Herausforderungen („coping“) hinauszugehen und den aktuellen Digitalisierungsschub als Transformationsprozess soziotechnischer Konfigurationen (Geels 2004, S. 899) im Bereich der universitären Lehre zu verstehen. Dabei wird es darauf ankommen, die Integration digitaler Technologien nicht allein vor dem Hintergrund ihrer didaktischen Eignung zu beurteilen, sondern als komplexen Veränderungsprozess zu erkennen, an dem eine breite Vielfalt strategischer Kalküle, kognitiver Ressourcen und technischer Ausstattungen beteiligt sind. Auf diese Weise wird eine fundierte Grundlage für den notwendigen Dialog und die Reflexion über mittel- und langfristige Entwicklungsmöglichkeiten angeboten.

Die empirische Basis dieses Vorhabens bilden zwei Projekte, in deren Rahmen Digitalisierungsprozesse während der COVID-19-Pandemie an vier steirischen Universitäten untersucht wurden. Die 64 durchgeführten Interviews mit verschiedenen Universitätsangehörigen wurden qualitativ ausgewertet. Dabei wurde Andreas Löschs (2013) Methode des Vision Assessments als Analysewerkzeug herangezogen, um fünf universitäre Zukunftsszenarien zu bilden, welche als Diskussionsgrundlage, nicht aber als Prognose der universitären Zukunft verstanden werden sollen.

## Konzeptualisierung soziotechnischen Wandels

Für die konzeptionelle Deutung der beobachteten Entwicklungen wird in diesem Beitrag der Ansatz der Multi-Level-Perspektive (MLP) herangezogen. Dieser von Franc Geels (2002) und Johan Schot (Geels und Schot 2007) entwickelte Ansatz nimmt technologische Veränderungsprozesse in den Blick und verdeutlicht, wie technische Entwicklungen mit sozialen Praktiken und strukturellen Voraussetzungen verbunden sind (Vorarbeiten dazu siehe Rip und Kemp 1998). Nur in ihrem Zusammenspiel kann man verstehen, wie sie zu dem führen, was soziotechnischer Wandel genannt werden kann (vgl. Smith et al. 2010).

Gemäß MLP können drei Ebenen unterschieden werden. Die mittlere Ebene – das soziotechnische Regime – beschreibt den Status quo (Geels 2004, S. 899). Sie klärt, in welcher Art und Weise Technologien und gesellschaftliche Verhältnisse in gegebenen Konfigurationen verflochten sind. Geels und Schot gehen davon aus, dass soziotechnische Regime durch eine Reihe von Faktoren stabilisiert werden, etwa gebaute Infrastrukturen, Handlungsroutinen, gesetzliche Regeln und soziale Normen. Die Grundfrage lautet, wie und wodurch sich existierende soziotechnische Regime ändern (Geels 2004, S. 899). Das geschieht gemäß der MLP durch Impulse von zwei Seiten: Von unten durch Nischeninnovationen und von oben durch disruptive Ereignisse auf übergeordneter Landscape-Ebene. Geels hält fest: „Transitions come about when dynamics at these three levels link up and reinforce each other“ (Geels 2004, S. 916, vgl. 2002, S. 1259 ff.; sowie Geels und Schot 2007, S. 399 ff.).

Dieser konzeptionelle Zugang lässt sich sehr fruchtbar auf den COVID-19-bedingten Digitalisierungsschub anwenden. An den österreichischen Universitäten wurden digitale Lehr- und Lerntechnologien vor dem Auftreten der Pandemie nur begrenzt eingesetzt, wie es in einem Interview formuliert wurde:

„Also, auch wenn es mir keiner glaubt, also das ist immer wieder so, ich sage: Ich baue daran. Ich baue daran. Also, diese [OER-]Plattform und so weiter, das sind genau diese Schritte, auch wenn mich der Rektor dreimal rausgehaut hat und uns gesagt hat: Das schaffen wir wieder ab. Heute ist der froh, dass wir das haben“. (Schlüsselperson, männlich)

Der überwiegende Teil der Lehre wurde im Präsenzmodus durchgeführt. Kommuniziert wurde Face-to-Face, verbal und in Echtzeit (Ebner et al. 2020; Rowies 2021, S. 2). Digitale Lehr- und Lerntechnologien können als Nischenprodukte verstanden werden. Sie wurden seit geraumer Zeit entwickelt, im universitären Kontext jedoch nur von wenigen Personen (vgl. Hamann et al. 2001) oder in Sonderfällen (vgl. Kim 2020) genutzt. Obwohl es Pionier*innen digitaler Lehr- und Lerntechnologien gab, welche Kompetenzen im Umgang mit diesen Nischenprodukten aufgebaut hatten, war das soziotechnische Regime der Präsenzuniversität bis zum Auftreten der Pandemie dominant und stabil (vgl. Geels und Schot 2007, S. 406).

Mit der Pandemie änderte sich diese Konstellation jedoch schlagartig. Das Auftreten von COVID-19 kann im Sinne der MLP als Landscape-Ereignis verstanden werden (Geels 2004, S. 914 ff.). Die infektiöse Erkrankung brach überraschend und krisenhaft über die Welt herein. In ihren Auswirkungen war und ist die Pandemie in hohem Maße disruptiv und hat globale Verwerfungen in nahezu allen Lebensbereichen nach sich gezogen. Die Bewältigung der Pandemie erforderte nicht zuletzt im Bereich der Bildung rasches Reagieren. Als Landscape-Ereignis im Sinne der MLP öffnete sich für digitale Lehr- und Lerntechnologien mit COVID-19 ein „Window of Opportunity“ (Geels 2002, S. 1262, 2004, S. 914), das Veränderungen erlaubte, die zuvor unter den Bedingungen des stabilen soziotechnischen Regimes der Präsenzuniversität nicht stattgefunden hatten bzw. auf Nischen beschränkt waren. Sowohl die disruptiven Veränderungen auf der Landscape-Ebene als auch die Existenz von Nischentechnologien mit den zugehörigen Infrastrukturen und Kompetenzen waren für die Bewältigung der Coronakrise wesentlich. Vor dem Hintergrund der gelungenen situativen Anpassung an die Herausforderungen der COVID-19-Pandemie kann nun nach den Bedingungen für eine dauerhafte Veränderung gefragt werden. Zur Beantwortung dieser Frage wird auf die MLP zurückgegriffen.

## Methode

Die analysierten Daten wurden im Rahmen von zwei Forschungsprojekten erhoben, die sich mit der pandemieinduzierten Digitalisierung an vier steirischen Universitäten befassten. Das Projekt „Reallabor – die eilige Digitalisierung“ erstreckte sich von Juni bis Dezember 2020. Diesem folgte ein weiteres zum Thema „Digitalisierungschancen steirischer Universitäten“, welches von Oktober 2020 bis Dezember 2021 durchgeführt wurde.^1^ Die Datenerhebungen wurden mit Methoden der qualitativen Sozialforschung durchgeführt (Flick et al. 2017; Strauss und Corbin 1996; Przyborski und Wohlrab-Sahr 2014). Insgesamt wurden 64 qualitative, leitfadengestützte Interviews mit universitären Lehrenden, Studierenden, administrativen Mitarbeiter*innen sowie Schlüsselakteur*innen der Digitalisierung geführt. In der Auswahl der Interviewpartner*innen wurde besonders auf Diversität geachtet, um eine große Vielfalt universitärer Lebensrealitäten abzudecken. Leitvariablen im Samplingprozess waren Geschlecht, akademische Disziplinen und Fachgebiete, administrative Bereiche, Senioritätslevels, hierarchische Positionen und Studienfortschritt. Zudem wurde Wert darauf gelegt, auch Erfahrungen von Personengruppen einzuschließen, welche von den pandemischen Auswirkungen in besonderer Weise betroffen waren, etwa Personen mit finanziellen Schwierigkeiten, Personen mit Behinderungen oder chronischen Krankheiten, Personen mit jungen Kindern und ausländische Personen mit Sprachbarrieren.

Die gesammelten Daten wurden inhaltsanalytisch mittels MAXQDA ausgewertet. Das Auswertungsverfahren orientierte sich dabei an der inhaltlich strukturierenden Inhaltsanalyse nach Kuckartz (2016, S. 101 ff.). So wurden über induktive Kategorienbildung ein übergreifendes Kategoriensystem und ein zugehöriger Kodierleitfaden gebildet, welche im Projektverlauf mehrmals überarbeitet wurden, um den sich ändernden pandemischen Bedingungen und deren Auswirkungen auf die Universität, welche sich im Datenmaterial abbildeten, gerecht zu werden. Anhand dieses Kategoriensystems und Kodierleitfadens wurde das gesamte Datenmaterial kodiert. Das finale Kategoriensystem wies dabei zehn übergreifende Hauptkategorien mit in unterschiedlichem Maße ausdifferenzierten Subkategorien auf.

Das kodierte Datenmaterial der übergreifenden Kategorie „Umstellung, Entwicklung, Vorbereitung“ mit besonderem Fokus auf die Subkategorie „Zukunft der Universitäten und der Digitalisierung“ bildet die Basis der vorgenommenen Analyse. Dem hohen Unsicherheitsgrad zukünftiger Entwicklungen Rechnung tragend, nimmt die vorgenommene Analyse von Zukunftsprognosen Abstand und versteht sich stattdessen als Vision Assessment (Lösch 2013; Lösch et al. 2021). Lösch betrachtet Visionen als „Mittel der Verständigung in der Gegenwart“, welche Diskussionen über soziotechnische Zukunftsvorstellungen, -wünsche oder -erwartungen prägen und gestalten (Lösch 2013, S. 12). Sein Konzept versteht Visionen als „strategisch ins Spiel gebracht[e]“ Narrative oder Beschreibungen der Zukunft durch einflussmächtige Individuen oder Kollektive, welche damit „Legitimation für bestimmte zukünftige Technologien und deren Innovationspotentiale für Wissenschaft und Gesellschaft erzeugen und beeinflussen“ wollen (Lösch et al. 2021, S. 338). Er bezieht sich damit unter anderem auf das Konzept der „sociotechnical imaginaries“ nach Jasanoff und Kim (2009) und betont die Notwendigkeit einer Interpretation von Visionen innerhalb ihres sozialen Kontextes (Lösch 2013, S. 11 f.; siehe auch Jasanoff und Kim 2009, S. 122 f.).

Das Ziel eines Vision Assessments ist es, Zukunftsvisionen zentraler Akteur*innen transparent zu machen (Lösch et al. 2021, S. 337). Dabei wird innerhalb des jeweiligen sozialen Kontextes die Umsetzbarkeit, Wünschenswertigkeit und Glaubwürdigkeit der Visionen vergleichend evaluiert (Lösch 2013, S. 11). Den „analytisch-explorativen Methoden“ Löschs folgend (Lösch et al. 2021, S. 339), wurden aus dem dargelegten, kodierten Datenmaterial fünf Szenarien herausgearbeitet (Lösch et al. 2021, S. 342). Die Bildung dieser Szenarien erfolgte induktiv und konzentrierte sich maßgeblich auf die Fragen, welche „Visionen“ oder Vorstellungen universitäre Akteur*innen von einer post-pandemischen Universität haben, wie sich diese Visionen aus Perspektive verschiedener Akteursgruppen unterscheiden und unter welchen Argumentationen und Narrativen die verschiedenen Szenarien von den Akteursgruppen als wünschenswert oder nicht wünschenswert beurteilt werden.

Ergänzend ist hinzuzufügen, dass die Analyse von Zukunftsszenarien nur eine von vielen Fragestellungen der genannten Projekte darstellte. Die dementsprechend breiter angelegte Erhebung umfasst daher Datenmaterial, das sich nicht allein auf Zukunftsvorstellungen digitalisierter Universitäten bezieht. Die identifizierten Szenarien sind somit keine vollständig ausformulierten Visionen einzelner Befragter, sondern idealtypische Konstrukte, die übergreifende Narrative und Erwartungen verschiedener Universitätsangehöriger reflektieren. So wurde auch die von Lösch beschriebene strategische Qualität von zukunftsorientierten Visionen nicht ausschließlich Entscheidungsträger*innen zugeordnet, sondern auf breitere universitäre Akteursgruppen ausgeweitet. Schließlich wurde Löschs Konzept auch dahingehend erweitert, dass es nicht nur vorrangig positive Zukunftsvisionen ins Auge fasst, sondern auch negative Szenarien inkludiert. Damit wird auch den Ergebnissen einer von Simone Eringfeld an der Cambridge University durchgeführten Untersuchung Rechnung getragen, dass Befragte negativ bewertete Zukunftsentwicklungen konkreter beschreiben konnten als positive Szenarien (Eringfeld 2021, S. 148). Auch Horváth et al. betonen, dass sowohl utopische als auch dystopische Zukunftsvorstellungen Einblick in geteilte gesellschaftliche Ideale und Ziele geben und damit die Aushandlungsprozesse der universitären Zukunft mitgestalten (Horváth et al. 2021, S. 12).

## Adaptionsfähigkeit und dauerhafter Wandel

Universitäten haben ihre Krisenfestigkeit insofern unter Beweis gestellt, als es ihnen gelungen ist, den Lehrbetrieb unter Bedingungen der Pandemie – insbesondere im Lockdown – aufrechtzuerhalten. Mit anderen Worten war es gelungen, das soziotechnische Regime der Präsenzuniversität temporär zu suspendieren (siehe etwa Ebner et al. 2020, S. 14 ff.; vgl. auch Geels 2007, S. 406 ff.). Diese gelungene Anpassungsleistung bedeutet jedoch nicht automatisch, dass situativ vorgenommene Veränderungen langfristig bestehen bleiben. Mithilfe der MLP kann nach den Bedingungen dauerhafter Veränderungen auf der Ebene soziotechnischer Regime gefragt werden. Franc Geels argumentiert, dass existierende technische Infrastrukturen und materielle Netzwerke den Status quo soziotechnischer Systeme stabilisieren (Geels 2004, S. 911). Neue Konfigurationen erfordern demgemäß neue technisch-materielle Ausstattungen. Darauf bezogen kann argumentiert werden, dass diese Voraussetzungen für eine dauerhafte Digitalisierung der universitären Lehre in hohem Maße gegeben sind. Digitale Lehr- und Lerntechnologien und die dazu erforderliche Infrastruktur wurden bereits vor der Pandemie erprobt (Bratengeyer et al. 2016, S. 15). Große Organisationen wie Universitäten haben die Voraussetzungen, um strategische Projekte (wie das Angebot digitaler Lehr- und Lernformen) zu betreiben, ohne dass diese den Zwängen kommerzieller Profite oder dem Diktat des Effizienzgewinns unterliegen (vgl. Weyer 2008). Diese Vorbedingungen waren für ein rasches Handeln wesentlich und ermöglichten (insbesondere während der mehrfachen Lockdowns) eine temporäre Umstellung auf eine nahezu vollständig digitalisierte Distanz-Universität. Seit dem Ausbrechen der COVID-19-Pandemie wurden diese technischen Infrastrukturen weiter ausgebaut und stehen dementsprechend auch weiterhin zur Verfügung. Ein weiterer Ausbau digitaler Infrastrukturen wie Cloud-Systeme, Aufzeichnungs- und Streamingtechnologien sowie Hardwareausstattungen wird in universitären Policy-Papers für die bevorstehende Entwicklungsperiode vorgesehen (Universität Graz 2021, S. 37 f.; Technische Universität Graz 2020, S. 13; Montanuniversität Leoben 2020, S. 34; Kunstuniversität Graz 2021, S. 77). Vor diesem Hintergrund sind die technisch-materiellen Voraussetzungen für einen dauerhaften digitalen Wandel durchaus gegeben. Weiters argumentieren wir, dass die interviewten Personen die Verfügbarkeit von Wissensressourcen für den Einsatz digitaler Technologien in der Lehre an der Universität als durchaus gegeben einschätzten. Zwar werden zwischen den Universitäten verschiedene Ausbaugrade wahrgenommen, doch darin keine prinzipielle Barriere für eine dauerhafte Digitalisierung der Lehre gesehen (siehe auch Lübcke et al. 2022, S. 88). Eine interviewte Schlüsselakteurin der Digitalisierung hält etwa fest:

„Wir haben da Life-Long-Learning – dadurch, dass die sehr viel im E‑Learning-Bereich natürlich schon anbieten für ihr Klientel, haben sie eine Expertin schon für E‑Learning und Mediendidaktik gehabt. [Name] selbst hat ja in seiner OE auch für Mediendidaktik zwei Expert*innen drinnen“. (Schlüsselakteurin der Digitalisierung, weiblich)

Wie Franc Geels argumentiert, sind für die Transformation soziotechnischer Konfigurationen darüber hinaus kognitive, normative und regulative Aspekte maßgeblich (2004, S. 904 f.). Geels bringt damit eine neo-institutionssoziologische Perspektive ein, mit der verdeutlicht werden kann, wie soziotechnische Netzwerke Regeln^2^ ausbilden, welche die Wahrnehmung und Handlungen ihrer Akteur*innen strukturieren (Geels 2004, S. 989). Dazu führt Geels weiter aus: „But human actors are not entirely free to act as they want. Their perceptions and activities are coordinated (but not determined) by institutions and rules“ (Geels 2004, S. 902). Außerdem wird diese Strukturierung von den Usern ihrerseits reproduziert (Geels 2004, S. 903).

### Kognitive Regeln

Wesentlich ist, wie Technologien und Praktiken Bedeutung zugeschrieben wird, wie Geels verdeutlicht: „*Cognitive* rules constitute the nature of reality and the frames through which meaning or sense is made“ (Geels 2004, S. 904). Hier geht es also darum, wie die Realität der Universität, ihre Aufgaben, Herausforderungen und die Rolle der Lehre durch kognitive Rahmen (Diskurse und Narrative) strukturiert werden. Vor diesem Hintergrund erweisen sich der regulatorische Rahmen und normative Überzeugungen als größere Hindernisse für einen dauerhaften soziotechnischen Wandel.

### Regulativer Rahmen

Im ersten Jahr der Pandemie wurden die geltenden Regeln für die universitäre Lehre suspendiert. Dies eröffnete ein „Window of Opportunity“ (Geels 2002, S. 1262) für die Verwendung digitaler Lehr- und Lernformen, wo diese zuvor eingeschränkt war. Ein regulativer Rahmen, der die Verwendung digitaler Technologien in der Lehre auch in Zukunft in breiter Weise möglich macht, wurde bislang nicht beschlossen. Zum Zeitpunkt der Erhebung waren Universitätsgesetz und Satzung der Universität nach wie vor am traditionellen Modell der Präsenzuniversität orientiert. So können Lehrveranstaltungen zwar mittels Video- bzw. Telekonferenz im digitalen Format durchgeführt werden, doch eine zeitliche Flexibilisierung durch das Verfügbarmachen aufgezeichneter Lehrinhalte wird derzeit nur als mögliche Ergänzung, nicht aber als Substitut für Kontaktstunden gewertet (Technische Universität Graz 2021, S. 19; Karl-Franzens-Universität Graz 2022, S. 14–15). Auch andere gesetzliche Regelungen, etwa im Bereich Datenschutz, müssen erst noch geschaffen werden, um die Ausschöpfung des vollen Potenzials digitaler Technologien möglich zu machen.

### Normative Überzeugungen

Aus Sicht der MLP sind normative Überzeugungen maßgeblich dafür, ob sich temporäre Veränderungen dauerhaft niederschlagen oder nicht. Geels verweist hier auf klassische soziologische Literatur: „*Normative* rules are often highlighted by traditional sociologists (e.g. Durkheim, 1949, Parsons, 1937). These rules confer values, norms, role expectations, duties, rights, responsibilities. Sociologists argue that such rules are internalised through socialisation processes“ (Geels 2004, S. 904).

Vor allem zu Beginn der COVID-19-Pandemie konnte auf große Motivationsreserven und hohe Veränderungsbereitschaft unter den Angehörigen der Universität zurückgegriffen werden. Die Bereitschaft zur Verwendung digitaler Lehr- und Lerntechnologien im Distanzmodus war selbst unter jenen sehr hoch, die sich diesen gegenüber skeptisch äußerten. Unterstützt wurde diese Dynamik durch Policy-Dokumente, die durch das Auftreten der Pandemie eine Chance zu längerfristigen digitalen Transformationen gegeben sahen (Technische Universität Graz 2020). Mittlerweile ist die Situation offensichtlich anders. Im zweiten Jahr der Pandemie war ein deutliches Bemühen zu beobachten, Studierende zurück an den Campus zu holen. In welcher Form digitale Technologien in der Lehre in Zukunft eingesetzt werden, bleibt unter den gegebenen Voraussetzungen vorerst offen. Weitgehend unklar ist nicht nur, wie die digitale Zukunft der Universität konkret aussehen soll, sondern ob es sich bei den jeweiligen Vorschlägen um wünschenswerte Szenarien handelt oder nicht (vgl. Lübcke et al. 2022, S. 26). Vor diesem Hintergrund wird das größte Hindernis für eine dauerhafte Etablierung digitaler Lehr- und Lernformen an der Universität im fehlenden Konsens über die normative Bewertung der digitalen Universität gesehen, welcher sich zentral im vorliegenden Datenmaterial niederschlägt. Diese grundlegend offene Situation nehmen wir zum Ausgangspunkt, um nach der digitalen Zukunft der Universität zu fragen.

## Zukunftsszenarien

Aus den erhobenen Interviewdaten konnten fünf Zukunftsszenarien herausgearbeitet werden. Zur analytischen Unterscheidung dieser Szenarien greifen wir auf die von Geels und Schot (2007) entwickelte Typologie soziotechnischer Regimeübergänge zurück. Wir argumentieren, dass die Szenarien imaginierter Zukünfte der digitalen Universität zwar mit Geels und Schots Typen der Transition verglichen und Parallelen aufgezeigt werden können, jedoch keine deckungsgleiche Entsprechung im Sinne einer direkten Zuordnung erfolgen kann. Dieser Schluss erfolgt erstens, weil im Kontext der vorliegenden Analyse Geels und Schots Typen soziotechnischer Regimeübergänge als idealtypische Extrempositionen verstanden werden müssen, und zweitens, weil die vorgestellten Zukunftsszenarien keine ausreichende Kohärenz und Konkretheit aufweisen, um eine trennscharfe Typisierung zu rechtfertigen. Meinungen und Positionen zur Zukunft der digitalen Universität sind heterogen, zum Teil unspezifisch, und basieren weitgehend auf den Erfahrungen, die in der Extremsituation der Coronakrise gemacht wurden. Dennoch zeigen sie, wie diese Szenarien von universitären Akteur*innen auf Basis unterschiedlicher Argumente und Narrative gestützt oder abgelehnt werden.

### Die Fernuniversität

Dieses erste Szenario imaginiert eine vollständig digitale Universität und wurde mit dem Bild der „Fernuniversität“ in Verbindung gebracht. In diesem Modell würden sämtliche angebotene Lehrveranstaltungen ohne physisches Zusammentreffen von Lehrenden und Studierenden abgehalten und vollständig in digitale Formate transformiert werden. Die potenzielle* Transformation *des aktuellen, stark auf Präsenzformate ausgerichteten Universitätsregimes hin zur nahezu vollständig digitalisierten Fernuniversität kann im Rahmen der MLP von Geels und Schot (2007) mit dem Typ der „*technological substitution*“ verglichen werden. Ausgelöst durch ein Landscape-Ereignis (COVID-19-Pandemie) wird das bestehende Regime unter Druck gesetzt und es eröffnen sich „Windows of Opportunity“ für in Nischen bestehende Innovationen (digitale Lehr- und Lerntechnologien). Sind diese Nischeninnovationen weit genug entwickelt und stabil, können sie das, unter dem Druck der Veränderungen in der Landscape-Ebene instabil gewordene, bestehende Regime ersetzen (Geels und Schot 2007, S. 409 ff.).

Das Szenario der Fernuniversität wurde von den Interviewpartner*innen deutlich abgelehnt. So wurde die Fernuniversität mit einer Reihe negativer Konsequenzen assoziiert. Die rein digitale Abhaltung, insbesondere bei Lehrveranstaltungen, in denen das Erlernen praktischer Fähigkeiten und Kompetenzen sowie kultureller und fachspezifischer Techniken im Vordergrund steht, wurde als höchst problematisch beurteilt. Weiters würde die Fernuniversität zu einer Entwertung des Universitätscampus führen. Mit dessen Verschwinden würden soziale Interaktionen und Kooperationen im Unterricht gefährdet oder gänzlich eliminiert werden (vgl. Bork-Hüffer et al. 2021, S. 9 f.). Damit wären auch die Möglichkeiten zur Netzwerkbildung drastisch reduziert. Auf sozialer Ebene wurden Vereinsamung und psychische Belastungen befürchtet, die auch zu einer Benachteiligung von Jungforscher*innen führen könnten (vgl. Horváth et al. 2021, S. 2). Besonders von Studierendenseite wurde die soziale Komponente des Campuslebens als motivationsfördernd und qualitätssteigernd hervorgehoben (vgl. Brannen et al. 2020, S. 14).

Auch wurde immer wieder auf die Bedeutung von Lehr- und Büroräumlichkeiten sowie Infrastrukturen des traditionellen Universitätscampus (z. B. Labor, Bibliotheken, Lernplätze) hingewiesen. Die Bedeutung der universitären Infrastrukturen für die Entwicklung von praktischen Fähigkeiten wird auch von Cunningham und Walton (2016) betont. Im Modell der Fernuniversität sehen die Befragten diesen Mehrwert, den ein Campus bietet, verloren. Zudem käme es aufseiten von Studierenden und Lehrenden zu erhöhten Kosten für die Anschaffung der erforderlichen technischen Ausstattung (vgl. Bork-Hüffer et al. 2021, S. 12; außerdem Ewing 2021, S. 43).

P1: „Ich glaube, generell das ganze Studium online stattfinden zu lassen, das fußt halt sehr viel auf Eigenregie und wenn man die nicht hat, funktioniert das glaube ich auch nicht.“

P2: „Das hat sicher einen Grund, warum es Fernunis gibt, die nur wenig Leute besuchen.“

P1: „Ja, also, dass sich das jetzt nicht so durchsetzt. Aber ich glaube auch, dass dann in Präsenz einfach mehr tatsächlich hängen bleibt.“ (Studierende, männlich (P1) und weiblich (P2))

Weiters wurde bedacht, dass eine ortlos gewordene Universität zwar ein internationales Publikum ansprechen könnte, doch zugleich mit Universitäten aus aller Welt konkurrieren würde.

„Was unterscheidet dann eigentlich so eine Uni noch von einem generellen Fernstudium, was macht eine Uni aus? Und zum Beispiel kann ich mir vorstellen, dass jetzt irgendeine Fernstudienuni, die irgendwie schon immer digital ist, dass die eigentlich sogar qualitativ hochwertiger sind als irgendeine [Universität], die da jetzt irgendwie innerhalb von zwei, drei Wochen digital hat umstellen müssen.“ (Studierender, männlich)

Umgekehrt stützten sich die (selten genannten) Argumente für einen höheren Grad der Digitalisierung auf Narrative der barrierefreien und offenen Universität. Universitäre Policy-Papers deklarieren die Universität als inklusive und diversitätsfördernde Einrichtung, die es berufstätigen oder internationalen Studierenden, Personen mit Betreuungspflichten, Behinderungen oder Erkrankungen ermöglicht, ein Studium zu absolvieren (Universität Graz 2021, S. 37 f.; Technische Universität Graz 2020, S. 13; Montanuniversität Leoben 2020, S. 34; Kunstuniversität Graz 2021, S. 77). Die Zugänglichkeit für vielfältige Gruppen von Studierenden soll durch den Einsatz digitaler Lehr- und Lerntechnologien und die damit einhergehende Flexibilität erleichtert werden. Diese Argumentation kann einerseits vor dem Kontext normativer Werte und Ziele (vgl. Eringfeld 2021, S. 148), andererseits vor dem strategischen Anspruch, neue Zielgruppen von Studierenden anzusprechen, interpretiert werden (vgl. European University Association 2021, S. 5 f.; Ewing 2021, S. 42 f.).

Für den überwiegenden Teil der befragten Universitätsangehörigen war die Vorstellung, die pandemieinduzierte Notfallsdigitalisierung in Gestalt der Fernuniversität zum Dauerzustand zu machen, jedoch geradezu dystopisch, was auch in der einschlägigen Literatur bestätigt wird (vgl. Eringfeld 2021, S. 151; Bork-Hüffer et al. 2021, S. 11). Hingewiesen wird zudem auf die potenzielle Entwertung von Abschlüssen (Brannen et al. 2020, S. 14), den erhöhten Arbeitsaufwand für Lehrende und Administration (Gurukkal 2020, S. 95 f.; St. Onge et al. 2021, S. 12 ff.), den Abbau von geistes- und sozialwissenschaftlichen Studienangeboten (Pagliarini 2021, S. 408), die Kürzung von Bildungsgeldern (Woicolesco et al. 2021, S. 3) und die generelle Abnahme der Lehr- und Studienqualität (Brannen et al. 2020, S. 14; Eringfeld 2021, S. 151 f.; Gurukkal 2020, S. 93 f.; Horváth et al. 2021, S. 139).

### Die traditionelle Präsenzuniversität

Das zweite Szenario – die traditionelle Präsenzuniversität – würde sich weiterhin auf die Durchführung von Lehrveranstaltungen und Prüfungen am Universitätscampus konzentrieren. Digitale Technologien würden wie vor der Pandemie Nischenprodukte bleiben und die eingesetzten didaktischen Konzepte würden fortgeführt werden, was insbesondere die Tradition des Frontalunterrichts im Vorlesungsformat (vgl. Lübcke et al. 2022, S. 7) einschließt. Die Universität würde weiterhin vorwiegend lokale Zielgruppen ansprechen und ihre Rolle als regionale Ausbildungsstätte wahrnehmen.

Im Kontext der MLP kann man die Rückkehr zurück zur prä-pandemischen Ursprungssituation mit Geels’ und Schots (2007) Typ der *Reproduktion *vergleichen. Eine solche Rückkehr zum alten Regime der traditionellen Präsenzuniversität wäre ohne großen Aufwand rasch umsetzbar (Geels und Schot 2007, S. 406 ff.), sobald durch die COVID-19-Pandemie kein Krisendruck mehr auf die universitäre Lehre ausgeübt wird. Obwohl die Vorteile der Präsenzuniversität für einige Lehrende und Studierende den Wunsch nach Rückkehr zur alten Präsenzuniversität begründen, zeigt die empirische Erhebung, dass eine Rückkehr zum unveränderten *status quo ante* mehrheitlich abgelehnt wird. Der überwiegende Teil der Interviewten betrachtete die Rückkehr zum traditionellen Präsenzbetrieb als Rückschritt. So wurde vor dem Hintergrund der neu gewonnenen Erfahrungen das Potenzial von digitalen Technologien erkannt und zu schätzen gelernt. Positiv bewertet wurde darüber hinaus, dass im Zuge der Pandemie innovative Unterrichtsformen ausprobiert wurden. Mit der Rückkehr zum traditionellen Präsenzunterricht würden viele dieser Initiativen obsolet werden und die neu erworbenen Kompetenzen verloren gehen.

„Da werden wir mit der Zeit gehen, weil wenn du nicht mit der Zeit gehst, dann geht die Zeit mit dir.“ (Schlüsselakteur, männlich)

Das Modell der traditionellen Universität wurde als negatives Szenario erachtet, wenn es als Rückkehr zu Lehr- und Lernformen betrachtet wurde, die als „antiquiert“ klassifiziert wurden. Digitalisierung wurde somit als Modernisierung und Mittel zur Qualitätssteigerung der universitären Lehre betrachtet. Vor diesem Hintergrund wurde die Rückkehr zur reinen Präsenzuniversität von den Befragten – wie schon die Fernuniversität – deutlich abgelehnt.

„Und ich glaube, das Schlechteste wäre, wenn man jetzt wieder darauf zurückgehen würde, also nur mehr alles in Präsenz zu machen. Finde ich, ist die falsche Richtung. Und genau die andere falsche Richtung ist, gar nichts mehr in Präsenz zu machen.“ (Lehrende, weiblich)

Das Szenario wurde als negativ bewertet, wenn es als Verschließen gegenüber notwendigen Reformen und Absonderung gegenüber der Außenwelt betrachtet wurde (vgl. Brannen et al. 2020, S. 12; Dumulescu et al. 2021, S. 1 f.; Bork-Hüffer et al. 2021, S. 21). Langfristig bestünde dann die Gefahr, hinter anderen Universitäten zurückzufallen. Nicht zuletzt würde ein strikter Präsenzmodus zur verstärkten Exklusion von berufstätigen und internationalen Studierenden sowie von Universitätsangehörigen mit Betreuungspflichten, Behinderungen, chronischen Krankheiten oder psychischen Problemen beitragen (vgl. Eringfeld 2021, S. 148).

Die Unterstützung von Szenarien, die der Universität vorrangig physische Präsenz einräumen, wird mit Narrativen argumentiert, die sich auf die Rolle der Universität als lokaler Bildungsträger beziehen. Das Argument artikuliert eher einen regionalpolitischen Bedarf, als es wirtschaftspolitische Ziele der Standortsicherung mobilisiert (vgl. Leydesdorff 2010). Zudem wird ein gewisses Eigeninteresse der Universität, die sich selbst nicht obsolet machen will, deutlich. Damit sind es eher endogene, auf die Universität selbst bezogene Aspekte, die hier artikuliert werden (vgl. De Freitas et al. 2015, S. 7).

### Die modulare Universität

Die modulare Universität beschreibt ein Szenario, in dem nicht mehr *alle* Lehrveranstaltungen der angebotenen Studiengänge „in-house“ durchgeführt werden würden. Nur *ein Teil* der für ein vollständiges Curriculum notwendigen Lehrveranstaltungen würde durch das lokale Personal angeboten werden. Studierende müssten demzufolge auf digitalem Weg Lehrveranstaltungen an anderen Universitäten absolvieren, um ein vollständiges Studium abschließen zu können. In diesem dritten Zukunftsmodell würde die Universität ihr Lehrangebot auf wissenschaftliche Spezialgebiete mit Alleinstellungsmerkmalen ausrichten. Lehrinhalte, die von ansässigen Spitzenforscher*innen nicht abgedeckt werden könnten, würden in digitaler Form zugekauft werden. Um ein internationales Publikum an Studierenden ansprechen zu können, würde ein wesentlicher Teil der Lehrveranstaltungen digital angeboten werden, während Präsenzunterricht vor allem zur Vermittlung praktischer Fähigkeiten, etwa im Zuge von Laborübungen oder Feldarbeit, eingesetzt werden würde. Das Ausbauen des digitalen Lehrangebotes würde zudem mit einer Förderung frei verfügbarer Lehr- und Lernmaterialien einhergehen, welche über Plattformen von Open Educational Resources (OER) angeboten würden.

Betrachtet man Geels’ und Schots (2007) Typologie der Transitionswege, so weist das Szenario der modularen Universität Elemente verschiedener Transitionstypen auf, insbesondere des „*reconfiguration pathway*s“: Geels und Schot beschreiben damit symbiotische, in Nischen entwickelte Innovationen, die im bestehenden Regime bei Bedarf zur Lösung von Problemen eingesetzt werden. Unter dem Druck der Landscape-Ebene führen diese Veränderungen mit der Zeit zu Veränderungen in der Grundstruktur des Regimes (Geels und Schot 2007, S. 411 ff.). Aufgrund der Outsourcing- und Digitalisierungsstrategien der modularen Universität müssten sich wesentliche Teile der Systemarchitektur deutlich verändern, während andere relativ unverändert bleiben könnten (weiterhin am Campus angebotene Lehrveranstaltungen). Weitere Parallelen bestehen in Bezug auf die Vielzahl an gleichzeitig eingesetzten, technologischen Innovationen (z. B. OER, Conferencing- und Streaming Software, digitale Prüfungssoftware), das Bestehen von (ausgewählten) Systemakteur*innen (Spitzenforscher*innen und -lehrende) sowie insbesondere auf die hohe Kompetitivität (internationaler Wettbewerb zwischen Universitäten, intensivierte Kompetitivität bei Stellenvergaben), die Geels und Schot als Merkmal des „reconfiguration pathway“ nennen (vgl. Geels und Schot 2007, S. 411 ff.).

Dem strategischen Charakter der modularen Universität entsprechend, wurde dieses Szenario von Personen in leitenden Positionen als potenzielle Strategie diskutiert, während andere Universitätsangehörige ein derartiges Zukunftsszenario nur selten in Erwägung zogen.

„Es gibt also Bildungsinstitutionen, die quasi vermehrt in diese Online-Kurse investieren und sagen: Okay, wir sind also die, die mehr oder weniger Provider sind. […] Und dann ist die Frage, wer spielt hart? Und wer spielt quasi Ausbildungsstätte?“ (Schlüsselakteur, männlich)

Die Argumente für eine stärkere Digitalisierung, insbesondere in Form der modularen Universität, rekurrieren vor allem auf Narrative des Wettbewerbs und des Effizienzgewinns. So würde die Umsetzung dieses Szenarios zu einer Qualitätssteigerung in der Lehre führen, da Lehrende in modularen Universitäten nur noch Lehrveranstaltungen in ihren Fachgebieten abhalten würden. Dies wurde wiederum als Möglichkeit betrachtet, die Reputation der eigenen Universität zu stärken und ein internationales Publikum an Studierenden, Forschenden und Lehrenden anzusprechen.

Es sind somit stark exogene Faktoren, die hier zur Begründung strategischer Erwägungen angeführt werden. Nicht ein selbstbestimmtes oder ideelles Ziel steht im Vordergrund, sondern eine mitunter als alternativenlos wahrgenommene Konkurrenzdynamik. Die Änderung dieser Umstände wird nicht als beeinflussbar wahrgenommen, stattdessen wird überlegt, wie es gelingen kann, im Wettbewerb mit anderen Universitäten und digitalen Bildungsplattformen zu bestehen.

„Verschiedene Universitäten liefern ja schon Content, das ist die Zukunft. Wir sind da noch am Anfang. […]. Die Gefahr besteht darin, zu erkennen, dass wir verschlafen haben. […] Ich frage mich, was bieten wir so Gutes an? Gibt es irgendwen auf der Welt, der [die referenzierte Universität] anklicken würde und dann irgendwas Tolles da erfahren würde auf dieser digitalen Basis?“ (Lehrender, männlich)

Die Entwicklungspläne der Universitäten beinhalten den verstärkten Einsatz von OER als etabliertes Instrument der universitären Lehre. Hierbei werden Kooperationen mit anderen Hochschulen für die Bereitstellung gemeinsamer Lehr- und Lerninhalte unter Verwendung digitaler Technologien und Plattformen angestrebt (Universität Graz 2021, S. 36 f.; Technische Universität Graz 2020, S. 11; Montanuniversität Leoben 2020, S. 34; Kunstuniversität Graz 2021, S. 73). In Ländern wie Neuseeland und Schweden sind modulare Curricula kooperierender Universitäten bereits stärker umgesetzt als in Österreich (vgl. French 2015, S. 2). Viele der antizipierten Vorteile einer modularen Universität, etwa die gesteigerte Flexibilität und Mobilität, das Ansprechen internationaler Zielgruppen sowie die Möglichkeit zu vielfältigen Studienangeboten durch Kooperation mit Partneruniversitäten, werden in der Literatur hervorgehoben (vgl. French 2015, S. 3 ff.; siehe auch Mohamed 2012, S. 245 f.). Risiken oder Nebeneffekte modularer Modelle, etwa ein erhöhter Arbeitsaufwand und fehlende Unterstützung für Lehrende sowie die Gefahr der Inkohärenz und Inkonsistenz in Qualität, Didaktik und Effektivität (vgl. French 2015, S. 6 ff.; siehe auch Olamo et al. 2019, S. 1379 f.), darüber hinaus auch grundlegende Barrieren internationaler Zusammenarbeit, etwa in Bezug auf Verständigung und Kommunikation (vgl. Mohamed 2012, S. 250 f.), wurden von den Beführworter*innen der modularen Universität jedoch kaum thematisiert.

### Die ressourcenoptimierte Universität

Das Szenario der ressourcenoptimierten Universität beschreibt ein viertes Universitätsmodell, in dem große Vorlesungen digital abgehalten werden würden. Die als interaktionsarm betrachteten Vorlesungen würden als asynchrone Lehrvideos angeboten werden – angeboten entweder durch die Universität selbst oder als zugekaufte, externe Ressourcen bzw. OER. Solche Aufzeichnungen könnten traditionelle Vorlesungen entweder vollständig ersetzen oder im Sinne von „Flipped Classroom“ als wesentlicher Teil des Unterrichts eingesetzt werden (vgl. Saichaie 2020, S. 98 f.). Die dadurch gewonnenen zeitlichen, personellen und finanziellen Ressourcen würden in Folge für interaktive Unterrichtsmethoden in den Präsenzlehrveranstaltungen eingesetzt werden. Dadurch würde, im Sinne der ressourcenoptimierten Universität, langfristig eine Verbesserung der Betreuungsdichte und Qualität der angebotenen Lehre eintreten.

Anders als im zuvor erläuterten Szenario der modularen Universität bliebe die ressourcenoptimierte Universität eine Volluniversität, die das gesamte curriculare Angebot selbst anbietet. Trotz dieses Unterschiedes kann auch die ressourcenoptimierte Universität mit dem Transitionstyp der Rekonfiguration gemäß Geels und Schot (2007, S. 411 ff.) verglichen werden. Im Sinne eines „reconfiguration pathway“ würde sich die ressourcenoptimierte Universität nach der Pandemie langfristig aus der traditionellen Präsenzuniversität herausentwickeln, um bestehende Systemprobleme (Überlastung, Betreuungsprobleme) zu entschärfen, und damit die Grundstruktur des Regimes mit der Zeit maßgeblich verändern (Geels und Schot 2007, S. 411). Wie schon bei der modularen Universität würden im Szenario der ressourcenoptimierten Universität neue, digitale Elemente mit solchen der Präsenzuniversität kombiniert werden. Beide Stränge würden eigenen didaktischen Konzepten folgen, jedoch weitgehend unabhängig voneinander durchgeführt werden. Die Zusammenführung würde demnach nicht innerhalb einzelner Lehrveranstaltungen erfolgen, sondern übergeordnet auf der Ebene der Lehrpläne. Aus diesem Grund lässt sich hier aus MLP-Sicht weniger von einer Transformation als vielmehr von einer Rekonfiguration sprechen. Darüber hinaus bilden das Fortbestehen von Systemakteur*innen im Regime und die potenziell hohe Kompetitivität unter den Anbieter*innen digitaler Lehrangebote (beispielsweise per OER) ebenso Parallelen zum „reconfiguration pathway“ (Geels und Schot 2007, S. 411 ff.).

Das Universitätsmodell der ressourcenoptimierten Universität wurde, im Kontrast zur modularen Universität, vor allem von Studierenden und Lehrenden imaginiert und als Lösung für bestehende Ressourcen- und Betreuungsprobleme betrachtet.

„Also ich persönlich hätte eigentlich nichts dagegen, wenn die VOs weiterhin wirklich nur online stattfinden, weil … ja, ich das wirklich ziemlich praktisch finde das Ganze. Ich finde aber trotzdem, dass schon so das ganze Semester lang irgendwelche Präsenzlehrveranstaltungen stattfinden sollten. […] Es gibt eh immer genug Labore und Übungen, und ich finde, wenn die halt präsent stattfinden würden, wäre das sicher nicht schlimm, wenn […] die VOs vielleicht, um das Ganze zu entlasten, einfach online stattfinden würden.“ (Studierende, weiblich)

Darüber hinaus wurde das Szenario mit mehr Flexibilität und damit einer verbesserten Studierbarkeit assoziiert.

„Also, Vorteile sind zum Beispiel die Aufzeichnungen, damit man jederzeit zu den Lehrinhalten zugreifen kann und die durch den Online-Betrieb resultierende Flexibilität.“ (Studierende, weiblich)

Aufgrund der als gering empfundenen Interaktivität des frontalen Unterrichtsstils in Vorlesungen brachten viele Studierende digitale Aufzeichnungen nicht mit einem Qualitätsverlust in Verbindung. Die asynchrone Verfügbarkeit böte hingegen Vorteile gegenüber reinen Präsenzveranstaltungen. Auch von Lehrenden wurden Vorlesungen häufig als ineffektiv und didaktisch veraltet empfunden (siehe auch Ewing 2021, S. 42).

„Ja, ich glaube, die Vorlesung ist sowieso ein interessanter Lehrveranstaltungstyp, das kann man gut online machen, mit der richtigen Software, da könnte man auch von woanders zukaufen und es sich ersparen, von Leuten, die das super machen. Also die Vorlesung ist sowieso ein antiquierter Lehrveranstaltungstyp, aus einer Zeit, wo es noch keine Bücher gegeben hat und einer liest vor.“ (Lehrender, männlich)

Umgekehrt wurde die Förderung von Präsenzlehrveranstaltungen mit verbesserten Betreuungsverhältnissen umfassend als positiv beurteilt, da sie mit erhöhter Interaktivität und didaktischem Mehrwert assoziiert wurden.

Insgesamt rekurriert das Szenario der ressourcenoptimierten Universität argumentativ stark auf Effizienzerwägungen. Die Argumentation legt nahe, dass digitale Formate ökonomisch und didaktisch gewinnbringend eingesetzt werden können, wo die Vorteile der traditionellen Präsenzlehre nicht mehr zur Geltung gebracht werden können (z. B. Massenvorlesungen). Diese freiwerdenden Ressourcen sollen dann kapitalisiert werden, indem sie in Gestalt verbesserter Betreuungsverhältnisse zur interaktiven Lehre in kleineren Gruppen eingesetzt werden. Mit diesen strategischen Kalkülen wird eine Perspektive gezeichnet, die mit der Zustimmung jener Universitätsangehörigen rechnet, die eine präsenzbasierte Lehre am Universitätscampus grundsätzlich vorziehen, dem Einsatz digitaler Technologien jedoch offen gegenüberstehen, sofern diese Vorteile für die eigene (Lehr‑)Tätigkeit eröffnen. Dieser individuelle Nutzen wird argumentativ über einen didaktischen Mehrwert (also auch für Studierende) verallgemeinert, welcher aus einer (inter‑)aktiveren und praxisorientierteren Unterrichtsgestaltung in kleinen Betreuungsverhältnissen hervorgehen soll.

Die erwogenen didaktischen Vorteile stehen im Einklang mit der Literatur: Bangerl und Gürtl gehen davon aus, dass die Erfahrungen mit digitaler Lehre während der Pandemie sowohl Studierende als auch Lehrende für die Bedeutung und Qualität interaktiver Lehre sensibilisierten (Bangerl und Gürtl 2021, S. 52 ff.; sowie Neuwirth et al. 2020). Qualitäten, die durch interaktive Lehr- und Lernmodelle entstehen können, sind etwa eine Steigerung des Interesses an den gelehrten Inhalten, die Förderung des Austauschs zwischen Lehrenden und Studierenden sowie eine erhöhte Motivation und Zufriedenheit der Interaktionspartner*innen (Sun et al. 2018, S. 77 f.). Kritisch wird das Modell der ressourcenoptimierten Universität allerdings insofern gesehen, als mitunter bezweifelt wird, dass sich mit aufgezeichneten Vorlesungen tatsächlich nennenswert Ressourcen einsparen ließen. Zudem wurde die didaktische Qualität rein asynchroner Lehre durchaus in Zweifel gezogen (vgl. Dumulescu et al. 2021, S. 2 ff.).

### Die digitalisierte Präsenzuniversität

Schließlich wird mit der digitalisierten Präsenzuniversität ein Zukunftsmodell beschrieben, in welchem digitale Technologien in die Präsenzlehre integriert werden würden. Darin würden digitale Technologien in verschiedenen Modellen der Unterrichtsgestaltung, etwa Hybrid Teaching, „Blended Learning“ oder „Flipped Classroom“, in die Präsenzlehre integriert werden.^3^

Durch die unmittelbare Verbindung von Präsenzlehre und digitalen Lehr- und Lerntechnologien innerhalb von konkreten Lehrveranstaltungen weist digitalisierte Präsenzlehre einen wesentlichen Unterschied zu den zuvor erörterten Szenarien auf. Weil digitale Elemente mit Formen der Präsenzlehre nicht bloß in separaten Lehrveranstaltungen realisiert, sondern integral miteinander verbunden werden, lässt sich das Szenario der digitalisierten Präsenzuniversität aus MLP-Sicht am besten mit dem „*transformation pathway*“ in Verbindung bringen.

Gemäß Geels und Schot (2007) tritt diese Form der Transition auf, wenn von der Landscape-Ebene Veränderungsdruck ausgeübt wird, Nischeninnovationen das bestehende Regime aber noch nicht zu ersetzen vermögen (Geels und Schot 2007, S. 406 ff.). Das Szenario der digitalisierten Präsenzuniversität beschreibt eine ähnliche Transition, die einerseits aus der Unzufriedenheit mit vollständig digitalen Lehr- und Lernformen (wie in der Fernuniversität) und den wahrgenommenen Problemen mit dem bestehenden System (der traditionellen Präsenzuniversität) resultiert, andererseits aber auch die wahrgenommenen Vorteile und Potenziale beider Modelle (z. B.: Flexibilität, didaktische Innovation, Qualitäten des Universitätscampus) aufzugreifen versucht. Im „transformation pathway“ wird die Entwicklung des Regimes durch das Einsetzen technischer Variationen von innen heraus modifiziert. Bei dieser Form der Transition kann neues Wissen im Regime aufgenommen werden, auch wenn Akteur*innen des alten Regimes weiterhin tätig sind (Geels und Schot 2007, S. 406 ff.). In didaktischer Hinsicht bietet dieses Szenario Möglichkeiten zur Fusion von Präsenzlehre mit digitalen Lehr- und Lerntechnologien wie etwa im Rahmen von Flipped Classroom oder Blended Learning (vgl. Saichaie 2020, S. 98 f.), setzen jedoch neue didaktische Ansätze und Kompetenzen voraus (vgl. Santos und Serpa 2017, S. 91 f.).

Das Szenario der digitalisierten Präsenzuniversität wurde von den Befragten als positiv beurteilt: Viele Studierende sahen in der digitalisierten Präsenzuniversität das wünschenswerteste Zukunftsszenario, ebenso sprachen sich Lehrende und universitäre Entscheidungsträger*innen dafür aus. Der Einsatz teildigitalisierter Lehr- und Lernformate führe langfristig zu einer aktiveren und nachhaltigen Wissensvermittlung. So ließen sich auf diese Weise Partizipation und Zusammenarbeit unter Studierenden fördern (vgl. etwa St. Onge et al. 2021; Morris 2014). Darüber hinaus wurde erwartet, durch hybride Lehrveranstaltungen Studierenden eine deutlich erhöhte Flexibilität zu bieten und so zur Inklusivität und Diversität der Universität beizutragen (vgl. Eringfeld 2021, S. 152 f.; siehe auch Ewing 2021, S. 42 f.).

„Wir müssen zurück, wenn die Pandemie vorbei ist, zur Universität, die im Blended Learning Ansatz das Beste aus den Welten mitnimmt. Es gibt wahnsinnig viele Vorteile von Präsenz, aber wenn ich neue Zielgruppen erschließen will, Studierende mit Kindern und Betreuungspflichten, Berufstätige, dann ist es ganz entscheidend, eine höhere Flexibilität zu schaffen. Das müssen wir mitnehmen.“ (Schlüsselakteur, männlich)

Die Vorstellung der digitalisierten Präsenzuniversität wurde von den Befragten als wertvoller Impuls zur bewussteren Unterrichtsgestaltung gesehen. So könnten als veraltet wahrgenommene Didaktiken und Lehrveranstaltungstypen durch die integrative Verwendung digitaler Technologien modernisiert werden. Damit stiege die Qualität der Lehre und die Möglichkeit zu mehr Flexibilität könnte geboten werden. Gleichzeitig bliebe die Qualität des Campus und seine Bedeutung als sozialer Ort erhalten.

„Ich hoffe, dass es bis zu einem gewissen Level digital bleibt, dass auch die Bereitschaft der Lehrenden bleibt, die Vorlesungsunterlagen den Studierenden zur Verfügung zu stellen und online oder im Hybridmodus zu unterrichten, also Präsenz und Online. […] Aber ich würde den Zugang zur Uni gerne wiederhaben und wirklich in der Uni Leute kennenlernen wieder, dass man wieder mit Leuten zusammensitzen, reden, gemeinsam für bestimmte Fächer lernen und so weiter, das wünsche ich mir schon, ja.“ (Studierender, männlich)

Vor allem didaktische Überlegungen wurden ins Feld geführt, wenn es darum ging, die Leistungen der digitalisierten Präsenzuniversität zu unterstreichen. Der kommunikative Austausch in Präsenz wurde als wichtiger Bestandteil qualitativ hochwertiger Lernprozesse herausgestrichen (vgl. Dumulescu et al. 2021, S. 2). Didaktische Erwägungen sprechen jedoch auch für den verstärkten Einsatz digitaler Technologien, einerseits dort, wo die Lehre in Form von Frontalvorträgen stattfindet oder hohe Teilnehmerzahlen vorherrschen, wo es ohnehin kaum zu Interaktionen kommt oder diese nur auf einige wenige Teilnehmer*innen beschränkt bleiben, aber andererseits auch als Bereicherung und Modernisierung der gesamten universitären Lehre, die im Kontext zunehmend digitalisierter Arbeits‑, Forschungs- und Lebensrealitäten aktuell bleiben muss.

Auch in der Literatur werden Lehrformate wie Blended, Flipped oder Hybrid Learning, die digitale und präsenzbasierte Elemente verbinden, mit didaktischen Vorteilen konnotiert, darunter verbesserte Lernergebnisse für diverse Gruppen von Lernenden und erhöhte Zufriedenheit von Studierenden und Lehrenden (Saichaie 2020, S. 97 f.).^4^ O’Byrne und Pythash geben allerdings zu bedenken, dass Lehrende und Studierende den Umgang mit derartigen Lehr- und Lernformen erst erlernen müssen, um die resultierenden Vorteile nutzen zu können, was wiederum Zeit und universitäre Unterstützung voraussetzt (O’Byrne und Pythash 2015, S. 139 f.).

Dennoch bleibt die konkrete Umsetzung und Umsetzbarkeit einer digitalisierten Präsenzuniversität vorerst unklar, etwa in Bezug darauf, welche Lehrveranstaltungen in Präsenz, vollständig digital oder hybrid sein würden oder welches digitale Equipment Studierende und Lehrende benötigen würden, um am Unterricht teilzunehmen (siehe auch Ewing 2021, S. 43). Digitalisierte Präsenzuniversitäten bräuchten leistungsfähige Infrastrukturen und personell gut ausgestattete Support- und Serviceeinrichtungen sowie Weiterbildungsangebote für techno-didaktische Unterrichtsgestaltung (vgl. Rubichi und Francescone 2019, S. 110 ff.; siehe auch Neuwirth et al. 2020; Saichaie 2020, S. 99; Bork-Hüffer et al. 2021, S. 22 f.). Hinzu kommt der Bedarf an rechtlicher Beratung.

## Schlussfolgerungen

Der vorliegende Artikel reflektiert den durch die COVID-19-Pandemie ausgelösten Digitalisierungsschub und wie sich dieser im Bereich der universitären Lehre niedergeschlagen hat. Die empirische Vorgehensweise stützt sich auf die Erfahrungen der Angehörigen vier steirischer Universitäten. Insbesondere wurde nach der Dauerhaftigkeit des kriseninduzierten Digitalisierungsschubes gefragt. Zur Beantwortung dieser Frage wurde auf die von Franc Geels (2004) ausgearbeitete Multi-Level-Perspektive zurückgegriffen. Mithilfe dieses analytischen Rahmens konnte gezeigt werden, dass wesentliche Voraussetzungen für einen dauerhaften Wandel des soziotechnischen Regimes der Präsenzuniversität hin zu einer digitalisierten Konfiguration bislang nicht vollständig gegeben sind. Zwar sind technische Infrastrukturen durchaus vorhanden und könnten mit entsprechendem finanziellem Aufwand rasch weiter ausgebaut werden^5^, doch fehlen (derzeit noch) eine Reihe anderer maßgeblicher Voraussetzungen für eine dauerhafte Etablierung des krisenbedingten Digitalisierungsschubes.

So erschwert der existierende Rechtsrahmen eine stärkere Implementierung digitaler Lehr- und Lernformen und priorisiert traditionelle Formate der Präsenzlehre. Große Uneinigkeit herrscht darüber hinaus hinsichtlich der Frage, wie die digitale Zukunft der Universität konkret aussehen soll. In den Worten der MLP: Es besteht „mis-alignment“, etwa weil die soziale Funktion des soziotechnischen Systems (noch) nicht klar ist (Geels 2004, S. 900, 913). Geels betont die Bedeutung normativer Überzeugungen für soziotechnische Transformationsprozesse. Wenn demnach unklar ist, warum ein Wandel stattfinden soll, welche Richtung konkret einzuschlagen ist und mit welchen Mitteln diese Zielvorstellung am besten zu erreichen ist, bestehen geringe Chancen, dass sich gegebene soziotechnische Konfigurationen dauerhaft verändern. Tatsächlich bestehen in exakt diesen Fragen divergente Vorstellungen unter den Angehörigen der Universität. Die durchgeführte Analyse verdeutlicht eine große Heterogenität unter den geäußerten Zukunftsvorstellungen (und den Einschätzungen zur Eignung digitaler Technologien, diese zu erreichen.).

Von den interviewten Personen wurden mehrere mögliche Formen in Erwägung gezogen, wie digitale Lehr- und Lerntechnologien künftig integriert werden könnten. So wurde das Szenario der Rückkehr zur Universität, wie sie vor der Pandemie verfasst war (die Präsenzuniversität) von dem Szenario der Fernuniversität kontrastiert. Beide Extrempositionen fanden wenig Zustimmung. Dementgegen sprachen sich einige Interviewpartner*innen für den Weg in Richtung modularer Universität aus, wo digitale Lehrinhalte zugekauft (oder per OER in Anspruch genommen) und nur spezialisierte Lehrveranstaltungen vor Ort in Präsenz angeboten werden würden. Positiv wurde weiters das Szenario der ressourcenoptimierten Universität gesehen, in welchem durch Digitalisierung hauseigener Lehrveranstaltungen (insbesondere großer Vorlesungen) eine Ressourcenumschichtung zugunsten besserer Betreuungsverhältnisse in Präsenz erwartet wird. Darüber hinaus wurde ein fünftes Szenario in Betracht gezogen, welches durch den integrativen Einsatz digitaler Technologien auf didaktischen Mehrwert in der Präsenzlehre abzielt. Die Uneinigkeit darüber, welches der fünf Szenarien zu bevorzugen sei, resultiert nicht zuletzt aus der Unterschiedlichkeit ihrer argumentativen Begründung.^6^ Hinter diesen Begründungsdiskursen stehen je divergierende normative Annahmen über die Ziele und Aufgaben der Universität. Wettbewerbsimperative stehen dem Streben nach Effizienzgewinn gegenüber. Daneben werden didaktische Argumente angeführt, aber auch Erwägungen gezogen, was notwendig ist, um als lokal verankerte Bildungseinrichtung nicht obsolet zu werden.

Die beobachtete Vielfalt an Sichtweisen zeigt vor allem, dass eine Konsensbildung zu normativen Überzeugungen darüber fehlt, in welcher Form und zu welchem Zweck digitale Technologien künftig in der universitären Lehre eingesetzt werden sollen. Aus Multi-Level-Perspektive (ist das Erfüllen sozialer Funktionen von soziotechnischen Systemen ein Resultat menschlicher Akteure. Fehlt die Einigkeit darüber, welche sozialen Funktionen erfüllt werden sollen, so) fehlt damit eine maßgebliche Voraussetzung für einen dauerhaften soziotechnischen Wandel (vgl. Geels 2004, S. 900). Zum gegebenen Zeitpunkt ist nicht klar, welches der identifizierten Szenarien in Zukunft am stärksten aufgegriffen werden wird, was auch daran liegt, dass eine Debatte darüber, ob und aus welchen Gründen die skizzierten Szenarien wünschenswert sind, bislang noch nicht geführt wurde. Nach wie vor ist die Einschätzung digitaler Lehr- und Lerntechnologien stark von ihrer Kontextualisierung durch die COVID-19-Pandemie geprägt. Digitale Technologien werden überwiegend als geeignete Maßnahmen zur Krisenbewältigung betrachtet – und darüber bestand vor allem zu Beginn der Pandemie ein breiter Konsens unter den interviewten Universitätsangehörigen. Nur selten wird ihr langfristiges Potenzial für die Gestaltung universitärer Lehre in Betracht gezogen. Daher kann auch nicht im Sinne Bijkers (1995) von einer Schließung der Debatte oder von einer Stabilisierung der soziotechnischen Konfiguration gesprochen werden. Der vorliegende Artikel kann mit dem vorgelegten Vision Assessment (Lösch 2013) jedoch einen Beitrag dazu leisten, die Betrachtung von empirisch erhobenen Zukunftsszenarien zur Grundlage von Aushandlungsprozessen mit möglichst vielen Akteur*innen bereitzustellen. Das Ergebnis dieser noch zu führenden Debatten und die Frage, wer tatsächlich eingeladen wird, an einer solchen Deliberation teilzunehmen (vgl. Lafont 2019; siehe auch Habermas 1981; und Jasanoff 2005, 2012) bleibt weiterhin offen.

## Footnotes

1 Das Projekt „Reallabor – die eilige Digitalisierung“ wurde vom Vizerektorat für Digitalisierung und Change-Management der Technischen Universität Graz gefördert. Das Projektteam setzte sich zusammen aus Bernhard Wieser, Viktoria Pammer-Schindler, Christian Dayé, Stefan Reichmann, Marion Rowies, Mia Bangerl, Franziska Gürtl und Kübra Karatas. Das Projekt „Digitalisierungschancen steirischer Universitäten“ wurde im Rahmen des Förderprogramms „Aus der Corona-Krise lernen“ des Landes Steiermark gefördert (ABT08-135342/2020). Das Projektteam setzte sich zusammen aus Stefanie Lindstaedt, Bernhard Wieser, Viktoria Pammer-Schindler, Stefan Reichmann, Mia Bangerl, Marion Rowies, Franziska Gürtl und Kübra Karatas.

2 Dazu hält Geels fest: „To avoid confusion between institutions and (public) organisations, I propose the general sociological concept of ‚rules‘ instead. Although one can quarrel about terms and exact definitions, it is more important to look at the general phenomena they aim to describe, i.e. coordination and structuration of activities“ (Geels 2004, S. 904).

3 Sowohl hybrides Lernen als auch Blended Learning weisen in der Literatur eine Reihe inhomogener Definitionen auf. Obgleich verschiedene Definitionen existieren, scheint die Mehrheit der Autor*innen hybrides Lernen als Unterrichtsmodell aufzufassen, in welchem Studierende einen Teil des Unterrichts im Lehrsaal oder Unterrichtszimmer und einen Teil des Unterrichts außerhalb des Lehrsaals verbringen, zumeist unterstützt durch digitale Technologien und in digitalen Szenarien (Saichaie 2020, S. 164; siehe auch O’Byrne und Pythash 2015, S. 137 f.). Der Begriff Blended Learning wird nach Saichaie als Kombination aus Präsenzlehre und Elementen (digital) mediatisierter Instruktion definiert, während Flipped Learning als Lernszenario beschrieben wird, in welchem Lernende sich mittels (digital aufbereiteter) Informationsquellen (z. B. Videos, Texte) auf Präsenzeinheiten vorbereiten können, um Partizipation und Verständnis zu fördern (Saichaie 2020, S. 98 f.). Eine Gemeinsamkeit aller drei Lerntypen bildet sich in der versprochenen didaktischen Wirksamkeit. Die Integration digitaler Elemente und digitalisierter Lernsegmente könne nach Shea et al. aufgrund zur Präsenzlehre komplementärer Qualitäten einen wesentlichen Mehrwert in der Lehre erzeugen (Shea et al. 2015, S. 539).

4 Saichaie weist aber auch darauf hin, dass die inkonsistenten Definitionen von Flipped‑, Blended- und Hybrid-Learning-Modellen empirische Probleme bedingen und mehr Forschung notwendig ist, um konkrete Vorteile definieren zu können (Saichaie 2020, S. 98).

5 Diese Aussage kann nur im Kontext des westlichen Bildungssystems getroffen werden. So weist Robert Arnove (2020) darauf hin, dass Schüler und Studierende in Entwicklungsländern während der Pandemie oftmals nicht am Unterricht teilnehmen konnten, da sie über keine passende technische Ausstattung verfügten oder in Gegenden lebten, wo kein WLAN oder nicht einmal Elektrizität verfügbar war (Arnove 2020, S. 43). Wie Blass (2003) betont, sind diese Perspektiven in (imaginierten und prognostizierten) Zukunftsmodellen häufig unterrepräsentiert (Blass 2003, S. 1047 f.). Die vorliegende Analyse bezieht sich daher allein auf das österreichische System der Hochschulbildung.

6 Ohne Zweifel können (und werden) auch andere Zukunftsmodelle imaginiert und diskutiert werden. Eine Übersicht von zentralen Elementen möglicher digitaler Universitätsmodelle (in Deutschland) geben etwa Lübcke et al. (2022, S. 30 ff.). Eine detaillierte Auflistung von Modellen, welche nicht aus den vorliegenden Daten ermittelt werden können, ist jedoch in diesem Rahmen nicht möglich.
